# *trans-*Resveratrol in Nutraceuticals: Issues in Retail Quality and Effectiveness

**DOI:** 10.3390/molecules171012393

**Published:** 2012-10-22

**Authors:** Damiano Rossi, Alessandra Guerrini, Renato Bruni, Eleonora Brognara, Monica Borgatti, Roberto Gambari, Silvia Maietti, Gianni Sacchetti

**Affiliations:** 1 Dipartimento Scienze della Vita e Biotecnologie-SVEB, UR7 Terra&Acqua Tech-Università di Ferrara, Corso Ercole I d’Este 32, 44121 Ferrara, Italy; 2 Dipartimento di Scienze degli Alimenti, Università di Parma, Viale G.P. Usberti 11A, I-43124 Parma, Italy; 3 Dipartimento Scienze della Vita e Biotecnologie, Sezione Biochimica e Biologia Molecolare, Università di Ferrara, Via Fossato di Mortara 74, 44121 Ferrara, Italy

**Keywords:** resveratrol, food supplements, dietary supplements, nutraceuticals, quality control

## Abstract

Fourteen brands of resveratrol-containing nutraceuticals were evaluated in order to verify their actual resveratrol content and to control if their health-promoting properties are related to manufacturing quality. Products included pure resveratrol capsules or multi-ingredient formulations with standardized amounts of resveratrol and other phytochemicals. Samples were analyzed for total *trans*-resveratrol, flavonoids, procyanidin, polyphenol content and the results were compared with the content declared on-label. Only five out of 14 brands had near label values, compliant with Good Manufacturing Practices (GMP) requirements (95–105% content of active constituent), four products were slightly out of this range (83–111%) and three were in the 8–64% range. Two samples were below the limit of detection. The greater the difference between actual and labeled resveratrol content, the lower was the antioxidant and antiproliferative activity strength. Dietary supplements containing pure *trans*-resveratrol exhibited a greater induction of differentiation towards human leukemic K562 cells when compared to multicomponent products. Great differences currently exist among resveratrol food supplements commercially available and GMP-grade quality should not be taken for granted. On the other side, dosages suggested by most “pure”, “high-dosage” supplements may allow a supplementation level adequate to obtain some of the purported health benefits.

## 1. Introduction

The supplementation of the Western diet with food-derived phytochemicals, in order to achieve intake levels otherwise unattainable with the standard diet, has become an established habit for health-conscious consumers, but also a powerful tool for marketing purposes. The global market for dietary supplements is growing faster than most other sectors in the food industry and epitomizes a cultural and scientific hybridization between food and medicines [1]. Such an overlap must be carefully scrutinized in order to prevent fraud or mystification and avoid false expectations that may be detrimental for the consumers and for the market. Marketing studies have highlighted a growing interest in health-promoting products enriched with active ingredients extracted from plants and foods, but the boundary between dietary supplements tablets and pharmaceutical pills is not always clear for consumers [2,3]. For instance, most food supplements are presented in a pharmaceutical form (pills, tablets, capsules) implicitly suggesting a pharmaceutical grade quality and and efficacy, something that is also evoked by producers through health claims and on-label information. It follows that consumers often believe that dietary supplements are not drugs and have fewer side effects than conventional medications, but they are also led to believe that quality and manufacturing is compliant with pharmaceutical regulations [3,4,5]. However, the legal background for pharmaceuticals and these special food products is different in most Western countries. Although these products may claim to provide a pharmaceutical-grade quality, dietary supplements are not required to be compliant with the same standards enforced for pharmaceuticals under the European laws (95–105% content of claimed active constituent) [1,6]. Contrarily to efficacy and toxicity of phytochemicals used for health purposes, the purity, stability and consistency of nutritional supplements are neglected issues and few studies provide surveys of their over-the-counter quality. The literature available for polyphenolic substances, although limited, underscores in particular a weak compliance between labeled content and the amount actually delivered in products sold in retail stores [7,8]. For instance, only the 50% of 40 food supplements containing anthocyanins were found to be true-to-the-label in terms of standardized content and for 12% of them the amount delivered to the consumer was negligible [8]. Similar data have been reported for isoflavone-containing dietary supplements [7]. Furthermore, a recent review summarized that “over 70 formulations of 25 different nutraceuticals revealed variable quality; no nutraceutical showed consistent high quality, but a number revealed consistent low quality” [9].

Resveratrol, commercially available both in pure form and in native or artificial combinations with other polyphenols, has enjoyed a skyrocketing commercial and scientific success since 1992 [10]. During the last two decades this stilbene derivative has been found both as a glucoside and as an aglycone in red wine, in *Vitis vinifera* L. berry skin and seeds, in *Polygonum cuspidatum* Sieb. et Zucc. roots and in few other edible sources like peanuts, pistachios, cocoa and hop [11]. It has been linked to a plethora of health beneficial effects, including antioxidant and antinflammatory relief, the putative prevention of DNA cleavage, prevention of cardiovascular diseases, diabetes and cancer and has established itself as a staple substance for anti-aging with the support of more than 4,000 scientific papers and reviews [12,13,14,15,16,17,18,19,20]. Only recently its *in vitro* and *in vivo* potential has been partially confirmed by the first clinical evidence of efficacy, including the positive mimicking of the effects of calorie restriction [8,14,16,17,18]. Contrary to other plant secondary metabolites, adequate levels of resveratrol are particularily difficult to obtain via diet, due to its limited and variable content in foods. Given also the relatively easy availability in a pure form of its more active isomer (*trans*-resveratrol), it represents an ideal candidate for nutraceutical purposes. Consequently, we decided to set up a specific method and undertake a screening of dietary supplements containing resveratrol and sold via the Internet for their anti-aging and antioxidant properties, in order to verify their active substance content at time of sale and to determine if their health-promoting properties are related to manufacturing quality.

## 2. Results and Discussion

One of the most relevant shortcomings in molecular nutrition applied to food supplements is the translation of results from clinical and nutritional trials—carried out with pure and carefully weighed compounds—to a commercial reality. The latter, in fact, consists of products administered at the end of a manufacturing chain in which the quality, handling and stability of products may affect the overall efficacy. Resveratrol possess an overall good stability, but may become unstable after light exposure and only its *trans* isomer has been consistently linked to health beneficial effect in pharmacological and clinical studies, while *cis*-resveratrol had only seen limited success as an antiplatelet agent [15,17,21,22].

After setting up a dedicated extraction and HPLC protocol, 14 different brands of resveratrol-containing food supplements were evaluated. The pool comprised both capsules of pure resveratrol or multi-ingredient formulations with standardized amounts of resveratrol and other phytochemicals (Table 1). Results are summarized in Table 2, in which a comparison between labelled and actual *trans*-resveratol content has been made. Data were also correlated to the suggested dose and price for each 200 mg of *trans*-resveratrol actually delivered. Such an amount was chosen, in absence of a recognized RDA, as the upper limit of the suggested effective human daily dosage emerging from the first trials available at present time or by dose translation from animal studies [16,19,23].

If compared with the declared labeled content, our results show that only five of the 14 tested supplements had near label values compliant with pharmaceutical-grade quality requirements (95–105% content of claimed active constituent). Three of them were FDA-authorized supplements produced under pharmaceutical GMPs. On the contrary, four products were slightly outside of this range (83–111%) and three other products were found to contain an amount of *trans*-resveratrol comprising between 8 and 64% of the labelled content. In the two samples in which *trans*-resveratrol content was not standardized by the manufacturer, its abundance was below the limit of detection of our method. True-to-the-label content and pharmaceutical-grade *trans*-resveratrol products seem thus to be partially lacking at present. A further consideration can be made for the price-for-value that these products can offer: there is no clear pattern relating the substantial *trans*-resveratrol delivered and the sale price, as one of the best-quality products (PC003, both in terms of purity and total amount), had a very affordable cost while on the contrary PC012, one of the worst-performing, is also one of the most expensive supplements. It must be noticed, however, that most capsules formulated exclusively with *trans*-resveratrol in high dosages (samples 1–6), despite being not always true-to-the-label in terms of purity, allowed one to obtain a daily dosage above 200 mg if administered as suggested by the producers. Consumers interested in gaining benefits from supplementation of their diet with *trans*-resveratrol should turn their attention preferably to this kind of products, rather than to products with a multicomponent philosophy, whose actual amount of *trans*-resveratrol was quite erratic and below the amount recently suggested as effective [16,19,23].

**Table 1:** Description and on-label information of 14 resveratrol food supplements purchased on line.

Sample	Description	Weight per capsule (mg)	Claims	Declared quality	Form
PC001	500 mg of *Polygonum cuspidatum* extract standardized to 50% trans resveratol	500	Antioxidant, against age-related diseases	FDA Pharmaceutical GMP	P
PC002	500 mg of *P. cuspidatum *extract standardized to 99% trans resveratol	500	Antioxidant, against age-related diseases	FDA Pharmaceutical GMP	P
PC003	500 mg of *P. cuspidatum *extract standardized to 99% trans resveratol	500	Antioxidant	FDA certificated	P
PC004	600 mg of *P. cuspidatum* extract standardized to 50% trans resveratol	600	Antioxidant, against age-related diseases	n.a.	P
PC005	500 mg of *P. cuspidatum* extract standardized to 99% trans resveratol	500	Antioxidant, against age-related diseases	n.a.	P
PC006	500 mg of *P. cuspidatum* extract standardized to 99% trans resveratol	500	Antioxidant, against age-related diseases	n.a.	P
PC007	300 mg of *trans*-resveratol from *P. cuspidatum *standardized to 99%	307	Antioxidant	FDA certificated	M
PC008	100 mg of *trans*-resveratol from *P. cuspidatum*, with added quercetin, ferulic acid, Vitamin D3	365	Antioxidant	n.a.	M
PC009	500 mg multi ingredient with extracts from *Vitis vinifera* seed, skins, wine, vitamin C. No standardization provided, but resveratrol mentioned in the ingredients		Antioxidant	n.a.	M
PC010	40 mg of resveratrol (no *cis/trans* specifications)	200	Antioxidant, against age-related diseases	n.a.	P
PC011	75 mg of resveratrol (no *cis/trans *specifications) from *P.cuspidatum*, with added red wine and grape seed extracts .	700	Antioxidant	n.a.	M
PC012	16 mg of resveratrol (no *cis/trans *specifications), with vitamin C, green tea and *Vitis vinifera* peel and seed extracts	1200	Antioxidant, against age-related diseases	n.a.	M
PC013	200 mg of resveratrol (no *cis/trans* specifications), with added grape seed and red wine extracts, quercetin	600	Antioxidant, against age-related diseases	n.a.	M
PC014	150 mg of resveratrol 75% *trans*, with added silymarin, curcuma and astragalus extracts	500	Antioxidant, against age-related diseases	n.a.	M

**Table 2:** T*rans*-Resveratrol abundance in 14 food supplements purchased online and actual amounts delivered to the consumers.

Sample	Form ^a^	Purity on-label	*Trans-Resveratrol* ^b^	Deviation	Posology	Actual dosage ^c^	Value per cps ^d^
		%	%	%	cps/die	mg	US Dollars
PC001	P	50	49.90 ± 1.94	−0.2	1	249.5	0.77 (0.6)
PC002	P	99	95.18 ± 1.79	−3.82	1	480.9	1.44 (0.58)
PC003	P	98	99.03 ± 1.59	1.03	1	506.5	0.73 (0.3)
PC004	P	50	46.21 ± 1.10	−7.58	1	227.2	0.37 (0.32)
PC005	P	99	63.82 ± 0.35	−35.53	1	322.5	0.93 (0.58)
PC006	P	99	99.07 ± 0.92	0.07	1	501	2.5 (1)
PC007	M	98	39.59 ± 0.81	−60	1	120	1.1 (1.84)
PC008	M	27	22.48 ± 0.36	−16.74	1	270.7	1.23 (0.9)
PC009	M	-	<0.4	n.a.	1	n.a.	n.a.
PC010	P	20	22.18 ± 0.98	11	2	55.5	0.2 (0.72)
PC011	M	10.7	9.17 ± 0.11	−14.3	2	128.5	0.22 (0.34)
PC012	M	1.3	<0.4	n.a.	4	n.a.	n.a.
PC013	M	33	14.92 ± 0.37	−54.79	2	180.8	0.67 (0.74)
PC014	M	15	15.05 ± 0.53	0,05	2	226	0.41 (0.36)

**Legend**: ^a^ P = Pure, M = Multi-ingredient; ^b^ obtained with an HPLC validated method; ^c^ calculated according to on-label dosage and suggested daily posology; ^d^ per 200 mg of actual trans-resveratrol, calculated according to actual number of capsules per package and price at the moment of purchase.

On the contrary, if an integration of the antioxidant protection is the main goal, multicomponent products seem to offer similar results when compared with lower-tier pure resveratrol supplements, mainly as a consequence of the consistent radical scavenging and antioxidant properties of polyphenols usually included in these formulations. However, higher-quality products with pharmaceutical-grade *trans*-resveratrol, like samples PC002, PC003 and PC006, still outperformed most competitors in both radical scavenging and antioxidant efficacy (Table 3). 

**Figure 1:**
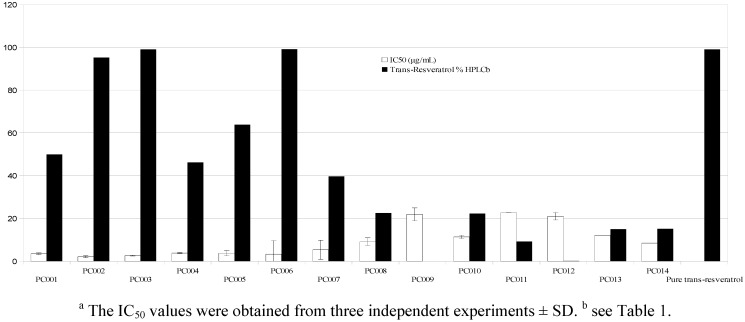
Effects of Resveratrol Commercial Product on Cell Growth of K562 Cells.

**Figure 2:**
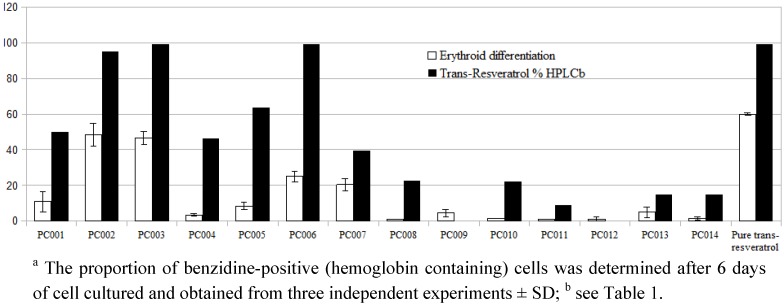
Effect of *trans*-resveratrol commercial products (10 µg/mL) on erythroid differentiation of human erythroleukemic K562 Cells ^a^.

*trans*-Resveratrol is also highly touted as a dietary chemopreventive agent and for such reason its use is often promoted in health claims. The available research has focused on a wide array of possible mechanisms for the putative prevention of cancer and a relevant number of *in vitro* models refers to the induction of differentiation and inhibition of proliferation in tumor cells [14,16,24]. In accordance with our philosophy, as laboratory data available for pure resveratrol should translate into evidences from commercially available products, the 14 food supplements were tested as antiproliferative and differentiating agents on human K562 leukemia cells (Figure 1 and Figure 2), a model in which *trans*-resveratrol has demonstrated its capability to inhibit proliferation and induce erythroid differentiation in a dose-dependent manner [25,26]. The antiproliferative assay evidenced good performance by supplements providing a *trans*-resveratrol concentration above 50%, while IC_50_ values scored by less concentrated samples were from 10 to 20 times higher (Figure 1).

**Table 3:** Total polyphenols, procyanidins, flavonoids and antioxidant properties of 14 resveratrol-containing food supplements.

Sample	Form ^a^	Tot. Polyphenols ^b^	Tot. Procyanidins ^c^	Tot. Flavonoids ^d^	IC50 DPPH	IC50 ABTS
			g/100g		mg/mL	
PC001	P	75.60 ± 2.94	2.28 ± 0.16	11.61 ± 0.52	16.34 ± 1.39	1.15 ± 0.07
PC002	P	93.72 ± 1.44	0.54 ± 0.08	0.00	13.15 ± 1.11	0.65 ± 0.05
PC003	P	98.47 ± 1.01	0.57 ± 0.04	0.00	14.40 ± 1.53	0.81 ± 0.05
PC004	P	77.86 ± 2.58	1.63 ± 0.15	9.81 ± 0.85	24.83 ± 1.79	1.37 ± 0.11
PC005	P	64.31 ± 2.34	0.44 ± 0.02	0.00	18.81 ± 1.81	1.19 ± 0.20
PC006	P	99.07 ± 1.05	0.39 ± 0.03	0.00	13.81 ± 0.99	0.77 ± 0.05
PC007	M	36.32 ± 3.63	0.33 ± 0.04	0.00	38.65 ± 2.04	1.55 ± 0.03
PC008	M	42.14 ± 2.84	0.37 ± 0.09	8.01 ± 0.36	20.23 ± 1.83	2.24 ± 0.07
PC009	M	13.80 ± 0.96	5.05 ± 0.25	1.78 ± 0.12	18.45 ± 1.03	6.77 ± 0.24
PC010	P	28.30 ± 1.94	0.79 ± 0.12	4.11 ± 0.21	25.65 ± 1.87	2.54 ± 0.19
PC011	M	21.71 ± 1.25	7.86 ± 0.86	3.13 ± 0.18	32.65 ± 1.43	3.71 ± 0.06
PC012	M	18.83 ± 1.14	3.33 ± 0.22	2.33 ± 0.16	16.93 ± 1.09	4.49 ± 0.03
PC013	M	51.18 ± 0.94	8.13 ± 0.91	17.23 ± 0.55	10.94 ± 0.93	1.79 ± 0.06
PC014	M	49.57 ± 1.84	0.58 ± 0.03	47.44 ± 1.56	18.51 ± 0.89	2.36 ± 0.08
*trans-Resveratrol*					17.9 ± 1.38	0.84 ± 0.05

**Legend**: ^a^ P = Pure, M = Multi-ingredient; ^b^ per 100g of capsule content, expressed as resveratrol; ^c^ per 100g of capsule content, expressed as cyanidin chloride; ^d^ per 100g of capsule content, expressed as quercetin.

**Figure 3:**
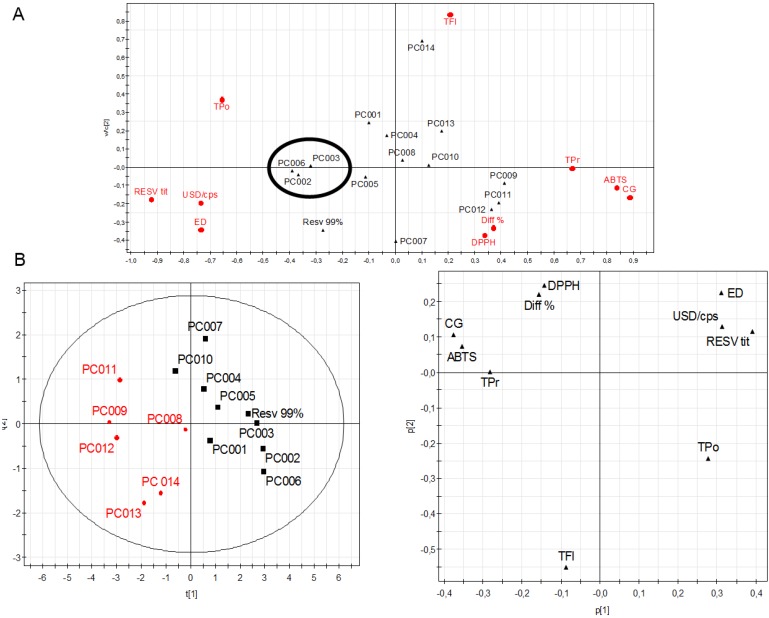
Score plot for PLS model and discriminant analysis.

**Figure 4:**
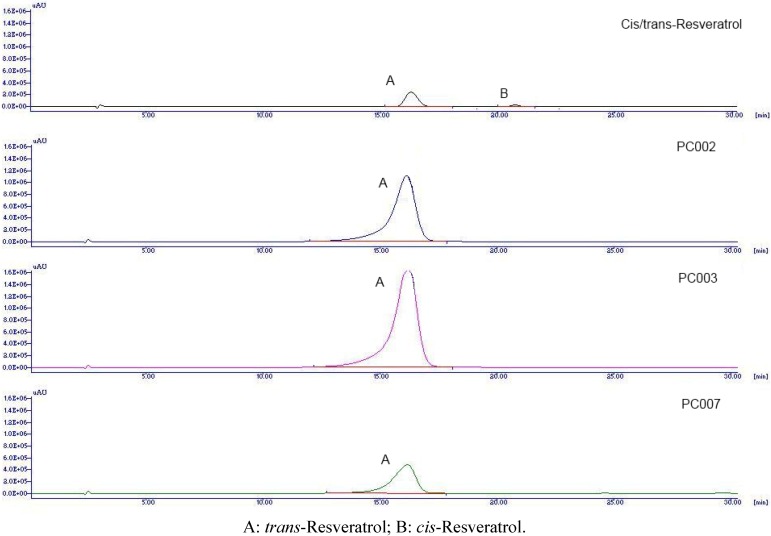
HPLC chromatograms of *cis/trans*-resveratrol, PC002, PC003 and PC007.

The results are consistent with data already available for *trans*-resveratrol. As far as the effects on differentiation are concerned, the data reported in Figure 2 demonstrate that supplements containing pure *trans*-resveratrol—albeit with different intensity due to a different substantial concentration—exibit a greater induction of differentiation towards benzidine-treated human leukemic K562 cells when compared to multicomponent products containing also, but not exclusively, *trans*-resveratrol. This *in vitro* model is very useful to study the molecular mechanism regulating the expression of embryonic and fetal human globin genes as well as to determine the therapeutic potential of new differentiation-inducing compounds [25,26]. The data in Figure 1 and Figure 2 suggest that in the commercial products in which resveratrol is present together with other bioactive molecules the effects on differentiation are very sensitive to the presence of compounds counteracting the effects of resveratrol stimulating erythroid differentiation.tatistical treatment of the collected data by PLS confirmed a good correlation between total polyphenols, total resveratrol, price and IC_50_ recorded for biological activities (Figure 3A). Moreover, the greater the difference between actual and labeled resveratrol content, the lower was the strength of the antioxidant and antiproliferative activities and the same trend was depicted for procyandin content. As confirmed by PLS-DA, samples PC002, PC003 and PC006 warrant the best activities at the higher price, while the lower price products have also a lower correlation to the different bioactivities (Figure 3B). PLS discriminant analysis outlined a clear separation in two classes, one composed by food supplements with resveratrol as the sole active ingredient and one constituted by multi-ingredient products (Figure 4). The first class provides a better and clear correlation with all of the bioactivities performed, suggesting the absence of a synergistic effect exerted by multiple ingredients. 

ABTS (2,2'- azino-bis(3-ethylbenzthiazoline-6-sulphonic acid) assay; CG, Cellular growth IC50; Diff%, percentage variance between labeled and actual resveratrol content; DPPH, (1,1-diphenyl-2-picrylhydrazyl) assay IC50; ED, Erhythroid differentiation IC50; Resv tit, actual amount of resveratrol by HPLC; Tfl, Total flavonoids; Tpo, Total polyphenols; Tpr, total procyanidins; USD/cps, price of each capsule in US dollars. Variables are represented with different filled symbols: triangle for samples, square for biological activities in score plot; square for food supplements with resveratrol as sole ingredient, reversed triangle for multi-ingredient products in discriminant analysis.

## 3. Experimental

### 3.1. Supplements

Three different production batches of 14 resveratrol-containing food supplements (Table 1) were purchased directly from online stores during 2010 and were analyzed before their expiry dates. For each package both the purchase price, dosages suggested and the number of capsules were noted, and then used to obtain the data reported in Table 2. Upon their arrival, products were stored at 2 ± 0.5 °C in the absence of light. Samples were anonymized and about 12 mg of the powder contained in the capsules was exactly weighted out, dissolved in 25 mL of ethanol, sonicated for 10 min at 25 °C and made up to volume.

### 3.2. Reagents and Standards

All the solvents used were of HPLC grade; formic acid, DPPH (1,1-diphenyl-2-picrylhydrazyl) bleaching test, ABTS (2,2'-azino-bis(3-ethylbenzthiazoline-6-sulphonic acid) were from Sigma-Aldrich (St. Louis, MO, USA). 99% *trans*-Resveratrol was from Merck (Darmstadt, Germany).

### 3.3. Resveratrol Quantification

The analysis were performed using a JASCO modular HPLC system (Tokyo, Japan, model PU 2089) coupled to a diode array apparatus (MD 2010 Plus) linked to an injection valve with a 20 μL sampler loop. The column used was a Tracer Extrasil ODS2 (25 × 0.46 cm, i.d., 5 mm) at a flow rate of 1.0 mL/min. The mobile phase consisted of solvent solution A (methanol) and B (water/formic acid = 95:5). The gradient system adopted was characterized by five steps: 1, isocratic, (30:70 v/v) for 15 min; 2, A raised progressively to (40:60) in 5 min; 3, A then raised to (60:40 v/v) in 30 min; 4, A achieved (80:20 v/v) in 5 min and 5, (100:0) in 5 min. Injection volume was 40.0 μL. The detector was set at 306 nm. The samples were dissolved in ethanol at a concentration 0.5 mg/ml. Quantifications were performed with an external standard method built on a calibration curve made with nine *trans*-resveratrol concentrations from 0.0008 to 1.2 mg/mL. Peak area was calculated by integration using Borwin ver. 1.22 (JMBS Developments, Grenoble, France). 

The method allowed the discrimination of *cis* and *trans*-resveratrol and was validated with the determination of Limit od Detection (LOD), Limit of Quantitation (LOQ), repeatability and recovery. The correlation coefficient r^2^ was 0.9998; LOD 0.5 μg/mL; LOQ 1.4 μg/mL. The recovery was estimated on PC009 sample by adding a known quantity of *trans*-resveratrol to reach a concentration of 0.30 mg/mL and resulted to be within 96–101%. Repeatibility, expressed as standard deviation at 100% of test concentration, was 1,09 µg/mL. A mixture of *trans*- and *cis*-resveratrol was prepared according literature [27]. A chromatogram of the solution of *trans*- and *cis*-resveratrol and of some representative samples is shown in Figure 4.

### 3.4. Total Procyanidins, Flavonoids and Polyphenols

The determination of the total polyphenolic, flavonoidic and procyanidin content in active extracts were performed using a ThermoSpectronic Helios-y spectrophotometer, according to previously described methods [28,29,30]. Total polyphenols are expressed as gallic acid, flavonoids as quercetin and procyanidins as cyanidin chloride.

### 3.5. Antioxidant Activity

Radical scavenging and antioxidant properties were performed in different assays, namely DPPH bleaching test and ABTS test according to previously described methods [31,32].

### 3.6. K562 Cells and Cell Culture Conditions

The human chronic myelogenous K562 cell line was obtained from the American Type Culture Collection (Rockville, MD, USA) and cells were cultured in RPMI 1640 medium (Sigma, St. Louis, MO, USA) supplemented with 10% fetal bovine serum (FBS; Celbio, Milan, Italy), 2 mM L-glutamine (Sigma-Aldrich), 50 U/mL penicillin, and 50 mg/ml streptomycin (Sigma-Aldrich) in humidified atmosphere of 5% CO_2_/air at 37 °C. 

### 3.7. Screening of Antiproliferative Activity

Antiproliferative assays were performed on K562 cells seeded at an initial concentration of 3 × 10^4^ cells/mL and cultured in the presence of increasing concentrations of dissoluted capsules or pure *trans*-resveratrol, used as positive control. Cell number/ml was determined after 3, 4, and 5 days, that is on the log phase of cell growth. Cell growth was studied by determining the cell number per milliliter with a ZF Coulter Counter (Coulter Electronics, Hialeah, FL, USA) [33]. 

### 3.8. Screening of Erythroid-Differentiation Activity

The proportion of benzidine-positive cells was determined after 4, 5 and 6 days of cell culture using a solution containing 0.2% benzidine in 0.5 M glacial acetic acid (10% H_2_O_2_ ) as elsewhere described [33]. 

### 3.9. Statistical Analysis

Principal component analysis (CA) and PLS-DA using “mean centering” as data pretreatment were conducted using the software SIMCA-P (v. 11.0; Umetrics, Umea, Sweden). The value of standard deviation is obtained from three independent experiments.

## 4. Conclusions

The reported differences among different brands of seemingly similar products legitimates the policy enforced by regulating agencies like EFSA, for which an health claim must be demonstrated for each specific formulation and not for a generic ingredient. In order to be properly effective outside of a trial unit, *trans*-resveratrol commercial products should meet quality standards comparable to those of chemically defined synthetic drugs and a strict quality control policy must be enforced. The number of formulated products exhibiting extreme deviation from label claims and the wide range of biological responses provided by products marketed under the same generic aegis of “resveratrol” is in fact particularly worrying. This leads to suggest that an erratic formulating quality may have a significant impact on their efficacy in humans. Moreover, pharmaceutical-grade production should not be taken for granted by regulators and consumers, whose critical appraisal to these products must be fostered. On the other side, it also proves that dosages suggested by most “pure” and “high-dosage” resveratrol supplements may allow users to achieve a supplementation level adequate to obtain some of the purported health benefits. A further consideration should be made on the fact that resveratrol has been proposed as a possible fetal hemoglobin inducer in sickle-cell anemia (SCA) and beta-thalassemia, despite the fact that its actual bioavailability is strongly influenced by human metabolism. As clearly shown in Figure 2, significant differences do exist between *trans*-resveratrol commercial products and ability to induce erythroid differentiation, a parameter often correlated with the clinical property to induce fetal hemoglobin production in erythroid precursor cells [34]. Accordingly, a careful analysis of these commercial products on HbF production is a required step before their possible use in clinical trials. On the other hand, the analysis of the effects of components present in formulated products exhibiting low levels of erythroid inducing activities and containing, in addition to resveratrol, other molecules might facilitate the identification of novel inhibitors of erythroid differentiation.
